# Chronic exposure to mercury increases arrhythmia and mortality post-acute myocardial infarction in rats

**DOI:** 10.3389/fphys.2023.1260509

**Published:** 2023-10-19

**Authors:** Keren A. S. Bello, Maria Clara B. Wilke, Rakel P. Simões, Maicon Landim-Vieira, Paulina Langa, Ivanita Stefanon, Dalton Valentim Vassallo, Aurélia Araújo Fernandes

**Affiliations:** ^1^ Department of Physiological Sciences of the Federal University of Espirito Santo, Vitória, Espirito Santo, Brazil; ^2^ Department of Biomedical Sciences, College of Medicine, Florida State University, Tallahassee, FL, United States; ^3^ Department of Medicine, Division of Cardiology, College of Medicine, University of Illinois at Chicago, Chicago, IL, United States

**Keywords:** mercury, arrhythmias, eletrocardiogram, myocardial infarction, mortality

## Abstract

**Introduction:** Mercury (Hg) is a heavy metal that causes a variety of toxic effects in eukaryotic cells. Previous studies have reported detrimental effects of mercury toxicity in the cardiovascular system. Given the importance of understanding the relationship between Hg and cardiovascular disease, we sought to investigate if the Hg could worsen the myocardial repercussions following ischemic injury. We demonstrated that once mercury toxicity is established, it can influence the outcome of myocardial infarction (MI).

**Methods:** Male Wistar rats received intramuscular injections of either saline (NaCl 0.9%) or mercuric chloride (HgCl_2_, first dose of 4.6 μg/kg, and subsequent doses of 0.07 μg/kg/day) for 4 weeks. Three weeks post-exposure, we induced transmural infarction in the left ventricle free wall through coronary artery occlusion surgery. Results: ECG recordings obtained from MI groups demonstrated alterations in the rhythm of the heartbeat/heart electrical activity, as expected, including ventricular extrasystoles and ventricular tachycardia. However, the MI group exposed to Hg (MI-Hg) exhibited augmented ventricular extrasystoles and ventricular tachycardia compared to the MI group. Also, Basckó coefficient revealed that the arrhythmic events—after MI—were aggravated by Hg exposure.

**Discussion:** Our results indicate that the significantly increased mortality in MI-Hg groups when compared to MI (21%, MI vs 32%, MI-Hg) is correlated with greater occurrence of arrhythmias. In conclusion, this study further supports the idea that exposure to mercury (Hg) should be recognized as a significant risk factor that exacerbates the impact of cardiac ischemic injury, potentially leading to an increased mortality rate among patients experiencing acute MI.

## 1 Introduction

Mercury (Hg) is a heavy metal that interferes with the activity of proteins involved in cardiovascular function. For example, there is a decrease in Sodium-Potassium ATPase, a reduction in Na^+^/Ca^2+^-exchange, a decrease in the sarcoendoplasmic reticulum (SR) calcium transport ATPase (SERCA-2a), and an increase in phospholamban protein expression. As a result, the Hg exposure can induce contractility dysfunction in isolated hearts (Furieri et al., 2011). The high affinity of Hg for sulfhydryl groups in amino acids, proteins, enzymes, and sulfur-containing antioxidants (such as N-acetylcysteine, α-lipoic acid, and glutathione) induces oxidative stress and mitochondrial dysfunction, which in turn affects calcium homeostasis in cardiomyocytes and hemodynamic performance (Houston et al., 2007; Branco et al., 2017; Genchi, 2017). Moreover, Hg reduces nitric oxide (NO) production, suppresses the inducible NO synthase gene expression, and induces the production of reactive oxygen species (ROS). In addition, Hg inactivates paraoxonase, an enzyme that prevents atherosclerosis, by decreasing the oxidation of LDL and acting in the reverse transport of cholesterol (Gonzalvo et al., 1997; Salonen et al., 1999; Azevedo et al., 2012; Balali-Mood et al., 2021).

Everyone in the world is likely exposed to some amount of mercury. In populations whose diet is based mainly on fish consumption, the risk of mercury exposure is increased (Genchi, 2017). In many developing countries, mercury is still a big problem. Efforts have to be made to reduce global mercury use. Exposure from ingestion of drinking water is a minor exposure pathway (www.atsdr.cdc.gov 2022 for Atsdr, 2022). However, it is concerning that an estimated 1 in 10 people (785 million) lack access to basic drinking water services, including 144 million who rely on untreated surface water (WHO, 2023). Between 2012 and 2015, the United States Geological Survey collected water samples from six locations in the US, revealing mercury levels ranging from 0.48 to 8.8 ng/L. The natural occurrence of mercury in the environment means that mercury is likely to occur in surface waters, even when anthropogenic sources of mercury are absent (Atsdr, 2022).

Previous studies have demonstrated that chronic exposure to Hg, even at low doses, is associated with increased systolic pressure, dysregulation of the renin-angiotensin system, hypertension, endothelial dysfunction, impaired vascular reactivity, and inflammation (Virtanen et al., 2005; Houston et al., 2007; Peçanha et al., 2010; Wennberg et al., 2012). Therefore, Hg exposure could be associated with the development of coronary artery disease, carotid atherosclerosis, and myocardial infarction (MI) (Salonen et al., 2000; Gallar et al., 2002). It has been demonstrated that chronic exposure to methylmercury in rat cardiomyocytes reduces cardiac rhythm, prolongs rate-corrected ventricular repolarization time, and increases ventricular repolarization dispersion. These parameters are associated with an increased risk of cardiac arrhythmia (Santos et al., 2020).

The literature is scarce on the dose and time dependent effects of Hg exposure to cardiac function. Indeed, the susceptibility to Hg toxicity differs among individuals and can be influenced by genetic factors (Jacob-Ferreira et al., 2010; Engström et al., 2011; Jacob-Ferreira et al., 2011; De Marcos et al., 2012; Barcelos et al., 2015).

Groups with high consumptions of fish, shellfish and marine mammals are likely higher exposure. However, these foods also offer various health benefits, creating a dilemma when it comes to determining their impact on wellbeing through consumption. Fetuses and infants are also vulnerable to effects from mercury, so pregnant women and recent mothers are also a concern for mercury exposure (Atsdr, 2022).

Exposures of infants *in utero* to these pollutants through maternal consumption of contaminated seafood can damage developing brains, reduce IQ and increase children’s risks for autism, Attention-Deficit/Hyperactivity Disorder. Adult exposures to methylmercury increase risks for cardiovascular disease and dementia (Atsdr, 2022).

The evidence linking mercury exposure to cardiovascular disease is extremely limited. Therefore, the acceptable levels of Hg in the blood for individuals with cardiac diseases or those who are susceptible to them should be thoroughly studied and clearly defined. This may entail the establishment of new regulations since this heavy metal contributes to increased cardiovascular risk in exposed individuals.

We aimed to assess whether the adverse effects of Hg exposure could exacerbate the consequences of MI. In the current study, myocardial ischemia was induced in rats by surgical ligation of the left coronary artery. Our results showed that the post-MI effects were worse in the Hg-exposed groups compared to non-exposed ones, which could be partially attributed to the increase of arrhythmic events, e.g., increased ventricular extrasystoles and ventricular tachycardia.

## 2 Methods

### 2.1 HgCl_2_ exposing

**FIGURE 1:**
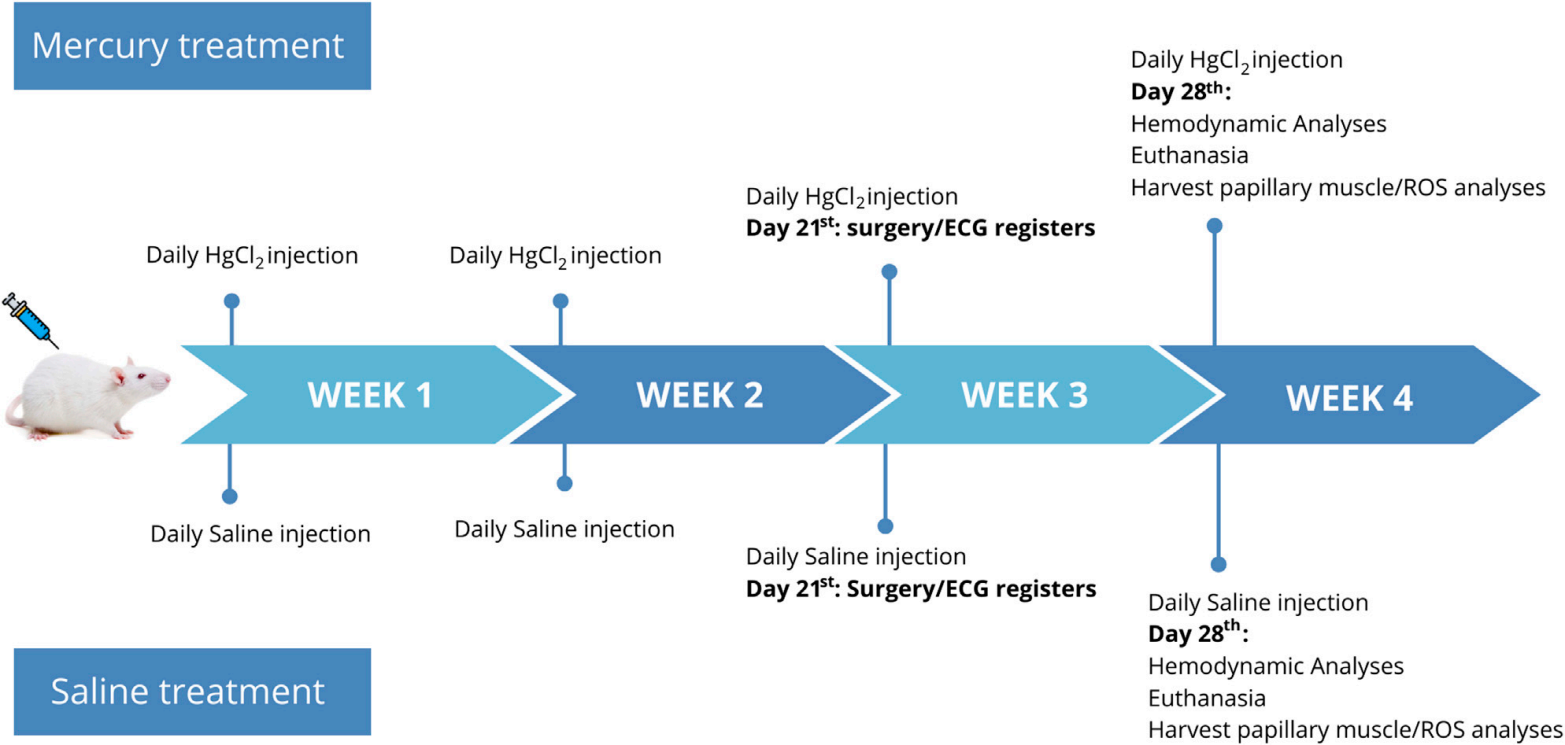
Timeline study: Week 1–4: animals were exposed to HgCl_2_ with an initial dosage of 4.6 μg/kg on the first day, followed by daily doses of 0.07 μg/kg, or received saline injections using the same dosages. Week 3: myocardial infarction (MI) surgery or sham surgery was performed. Electrocardiogram (ECG) recordings were conducted 5 min before and during 20 min after the surgery. Week 4: hemodynamic analyses were performed, followed by euthanasia of the animals. The papillary muscles were harvested for subsequent reactive oxygen species (ROS) analyses.

Twelve-week-old male Wistar rats (*Rattus novergicus albinus*) (230–250 g) were housed in cages under controlled temperature (20°C–25°C), light/dark cycle (12:12 h), and free access to filtered tap water and rat chow. The use and care of these animals were in accordance with ethical principles of animal research. Experimental protocols were approved by the Ethics Committee at UFES (Universidade Federal do Espírito Santo; protocol number 20/2018 and 24/2020). The animals were randomly distributed to receive saline (NaCl 0.9%—0.1 mL/300 g of body weight) or HgCl_2_ through intramuscular injection (first dose of 4.6 μg/kg, subsequent doses of 0.07 μg/kg/day) for 4 weeks, as shown in Figure 1. The dosage was adjusted daily aiming to reach the final plasma concentration of ∼20–29 nM by the end of the 4 weeks. The blood levels at the end of treatment correspond to 7.9 ± 0.59 ng/mL, as previously reported by Wiggers et al. (2008). Defining safe levels of mercury in blood continues to be an active research area. In 2000, the National Research Council of the National Academy of Sciences determined that a level of 85 μg/L in cord blood was associated with early neurodevelopmental effects.

The National Health and Nutrition Examination Survey (NHANES) findings from 2015 to 2016 revealed that the estimated geometric mean total blood mercury level in the adult U.S. population was 0.810 μg/L (CDC 2019). In the same year, National Health and Nutrition Examination Survey data indicated that total blood mercury levels in children aged 1–5 years were below the detection limit of 0.28 μg/L (Atsdr, 2022).

### 2.2 Coronary artery ligation

Rats that received either HgCl_2_ or saline for 3 weeks were randomly distributed into Sham operated or MI groups. The MI procedure was performed according to previously described methods (Fernandes et al., 2015). Briefly, the rats were anesthetized with ketamine (50 mg·kg^−1^) and xylazine (10 mg·kg^−1^) and underwent left lateral thoracotomy between the fourth and fifth intercostal spaces. Following exteriorization of the heart, the left atrium was pushed aside and the left coronary artery was ligated with 6.0 mononylon thread between the exit point of the pulmonary artery and left atrium. Next, the heart was returned to the thorax and the incision was closed with 1.0 cotton sutures. The procedure was performed to induce transmural infarction between 40% and 60% of the left ventricular surface without damaging the interventricular septum (Fernandes et al., 2015). MI was confirmed through ECG, which revealed typical findings such as ST segment elevation, QRS duration prolongation, changes in QRS amplitude, and shifts in the electrical axis of the QRS complex. Weight-matched rats were selected as controls and underwent all surgical procedures, excluding coronary ligation. These rats comprised the sham group, which received either saline (Sham) or HgCl_2_ (Sham-Hg).

### 2.3 Electrocardiogram (ECG) analysis

The ECG recordings were done 5 min before and 20 min after the MI surgery. A preamplifier (BioAmp, AdInstrumens, Australia) connected to a system of data acquisition (PowerLab, AdInstruments, Australia) was used to record the ECG parameters. ECG findings in MI include prolongation of the QRS duration, a decrease/increase in QRS amplitude, a shift in the electrical axis of the QRS complex, and ST segment elevation. These findings could be either associated or isolated to confirm the MI within the first 20 min of ECG recordings.

Ventricular arrhythmias were analyzed according to guidelines in the Lambeth conventions (Walker et al., 1988). The authors emphasized the definition, classification, and quantification of ventricular fibrillation (VF), ventricular extrasystoles (VE) and ventricular tachycardia (VT). The parameters measured in the present study were heart rate (HR), expressed in beats per minute (bpm); number of ventricular extrasystoles (VE), expressed in units (uni); duration of ventricular tachycardias (VT) and atrioventricular blocks (AVB), both expressed in minutes (min); and the score of Baczkó (Table 1). All analyses were done using D1 derivation.

**TABLE 1:** Arrhythmia score used to evaluate the incidence and duration of arrhythmias by giving a grade to each animal (Baczkó et al., 1997).

Arrhythmia	Score
No arrhythmias	0
VT < 10 s or other types of arrhythmias	1
VT 11–30 s, or other types of arrhythmias, no VF	2
VT 31–90 s or other types of arrhythmias, no VF	3
VT 91–180 s or other types of arrhythmias, or <10 s reversible VF, or both	4
VT > 180 s or other types of arrhythmias, or >10 s reversible VF	5
Irreversible VF	6

s, seconds; VT, ventricular tachycardia; VF, ventricular fibrillation.

### 2.4 Hemodynamic measurements

During the fourth week, which corresponds to the post-MI period, the surviving rats continued to receive either saline or HgCl_2_ injections. Subsequently, they were divided into the groups proposed in this study: Sham, which received saline; Sham-Hg, which received HgCl_2_; MI, which received saline; and MI-Hg, which received HgCl_2_.

At the end of the fourth week, the rats were once again anesthetized (with ketamine: 50 mg/kg, ip, and xylazine: 10 mg/kg, ip) and subjected to hemodynamic analyses.

Arterial blood pressure was measured, and ventricular measurements were achieved using a polyethylene catheter (P50) connected to the data acquisition system (MP100 Biopac Systems, Inc.; CA, United States). Briefly, the catheter was inserted in the right common carotid artery after its dissection. With the catheter placed in the artery, the stabilization was reached and were measured: heart rate (HR), systolic blood pressure (SBP) and diastolic blood pressure (DBP). Following, the catheter was advanced into the left ventricle (LV) to measure the left ventricle systolic pressure (LVSP), left end-diastolic pressure (LVEDP), and positive (+dP/dt) and negative (−dP/dt) rates of pressure development in the LV. Immediately after, the rats were euthanized, and the organs were harvested and weighed. The institutional guidelines strongly advise using three times the anesthesia dose for euthanasia. However, an overdose of anesthesia was administered, with three times the recommended dose of ketamine (150 mg/kg) and xylazine (30 mg/kg). The animals’ reflexes were tested before euthanasia, and euthanasia was performed once their pain reflexes were absent.

### 2.5 Scar size measurements

After harvesting and weighing the hearts, scar areas were measured as previously described by our group (Stefanon et al., 1994; Fernandes et al., 2015). The scar tissue was separated from the remaining left ventricle. The infarct size was determined by planimetry and expressed as a percentage of the left ventricle surface. The scar area was calculated using the Image J program (National Institutes of Health, EUA).

### 2.6 Reactive oxigen species analysis

Cardiac muscle samples were used for fluorescence analyses. Posterior papillary muscles from the left ventricle were dissected and the tissues were immersed in Krebs-HEPES buffer solution with 30% sucrose for 1 h. Then, they were embedded and immediately frozen with OCT (Tissue-Tek O.C.T.) and kept at −80°C until further analysis. The cardiac muscles were cut by cryostat into 4 μm-thick sections and placed on glass slides.

To evaluate *in situ* O_2_^−^ production, the oxidative fluorescent dye dihydroethidium (DHE) staining was applied, as previously describe Wiggers et al. (2008). Dihydroethidium freely permeates cells and is oxidized in the presence of O_2_^−^ to ethidium bromide, which is trapped by intercalation with DNA. Ethidium bromide is excited at 546 nm and emits at 610 nm. Frozen tissue segments were cut into 10 µm-thick sections and placed on glass slides. Serial sections were equilibrated under identical conditions for 30 min at 37°C in Krebs-HEPES buffer (in mM: 130 NaCl, 5.6 KCl, 2 CaCl_2_, 0.24 MgCl_2_, 8.3 HEPES, and 11 glucose, pH = 7.4). Then the buffer was drained. Subsequently, incubation was performed with a solution containing HEPES and DHE (2 µM) with or without Tiron for 30 min in a humidified chamber protected from light at 37°. Finally, the samples were captured using an inverted fluorescence microscope (Leica DM ×2500, ×40 objective) and with a Leica DFC 310 FX camera. The same image adjustments were applied to all groups. Fluorescence was detected with a ∼500–560 nm filter, and quantification was performed using Image J.

To evaluate *in situ* NO production, we used the oxidative fluorescent dye diaminofluorescein (DAF), as previously describe (Fardin et al., 2019). The slides were incubated for 30 min at 37°C in a phosphate buffer (0.1 M) containing CaCl_2_ (0.45 M). Then, the buffer was drained, and the excess was dried. Subsequently, the incubation was performed with a solution containing 4,5-diaminofluorescein (8 μM DAF-2) diluted in a phosphate buffer solution with CaCl_2_, and DAPI (4′,6-Diamidino-2-phenylindole dihydrochloride -DAPI, 1:10,000), and L-NAME (100 µM) or without, for 30 min in a humidified chamber protected from light at 37°. Next, the slides were imaged with an inverted fluorescence microscope (Leica DM ×2500, ×40 objective), with a Leica DFC 310 FX camera. The same image adjustments were used in all groups. The fluorescence was detected using a ∼500–560 nm filter. Three to five images were analyzed from each animal. The images were extracted from different parts of the posterior papillary muscles, and quantification was performed using Image J software.

### 2.7 Data analysis

Results are presented as mean ± SD. Continuous variables were compared by 2-way ANOVA followed by the Tukey test to determine differences among groups (Sham, Sham-Hg, MI and MI-Hg). Comparisons between MI groups aiming to compare the scar size were made using the Student’s t-test. *p* values < 0.05 were considered significant.

## 3 Results

### 3.1 Scar area

**FIGURE 2:**
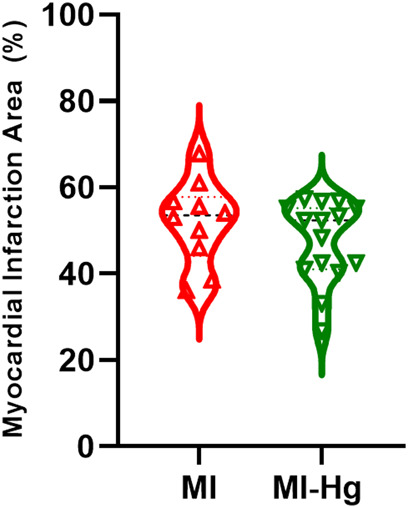
Scar size of myocardial infarction (MI) compared to the total area of the LV, expressed as a percentage (%). Data are expressed as mean ± SD values. For statistical analysis, the unpaired Student’s t-test was used, values of *p* < 0.05 were considered significant (MI = 51.87 ± 9.69%; MI-Hg = 47.62 ± 9.46%; *p* = 0.96).

The mean scar size was 51.87% ± 3.07% in the MI group and 47.62% ± 2.44% in the MI-Hg group (Figure 2), both falling within the desired range of 40%–60%. This range represents a moderate to large myocardial infarction, which allows for the evaluation of this variable in a relatively homogeneous group of animals, while excluding factors associated with scar size area.

### 3.2 Survival rate and correlation of mortality

**FIGURE 3:**
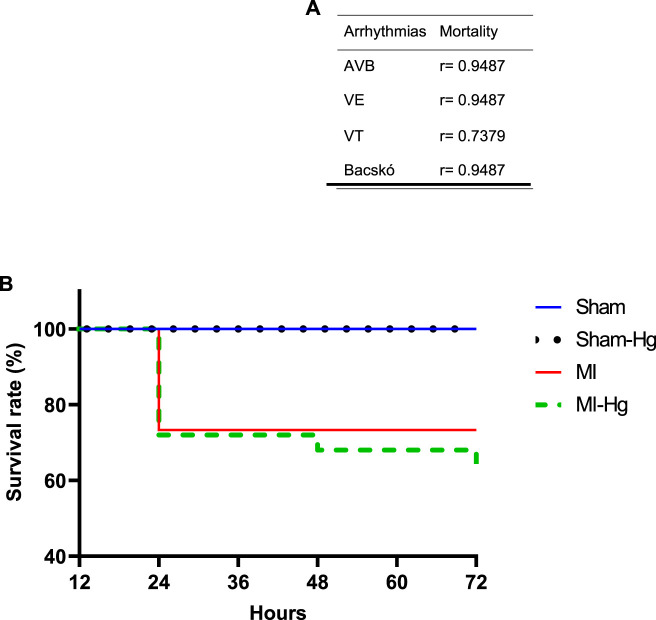
Survival rate and correlation of mortality. **(A)** Correlations between mortality and atrioventricular blocks (AVB), ventricular extrasystoles (VE), ventricular tachycardia (VT), and Baczkó coefficient were detected through the Spearman’s test. **(B)** Probability of survival 24, 48, and 72 h after surgery. Data are expressed in percentage (%). Sham (*n* = 25) = 100% of survival; Sham-Hg (*n* = 29) = 100% of survival; MI (*n* = 14) = 78.57% of survival; MI-Hg (*n* = 22) = 68.18% of survival. Differences were analyzed using the Kaplan-Meier test. *p* < 0.05 was considered significant.

Survival evaluation showed that the group infarcted and exposed to Hg (MI-Hg) had a survival rate of 68.18% and mortality rate of 31.82%. Meanwhile, the infarcted group not exposed to Hg (MI) had a survival rate of 78.57% and mortality of 21.43% (Figure 3B). Strong correlations between mortality and atrioventricular blocks (AVB) (*r* = 0.9487), ventricular extrasystoles (VE) (*r* = 0.9487), ventricular tachycardia (VT) (*r* = 0.7379), and Baczkó coefficient (*r* = 0.9487) were detected through the Spearman’s test (Figure 3A).

### 3.3 Electrocardiogram recordings

**FIGURE 4:**
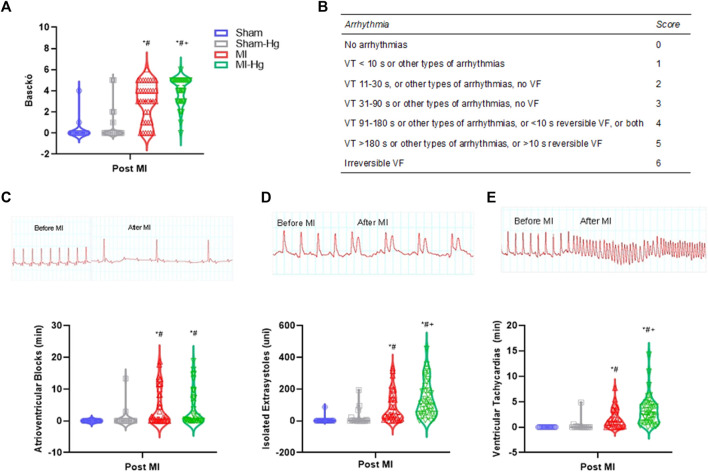
The effect of Hg on rhythm before and after surgery of myocardial infarction. ECG recordings from LabChart program. **(A)** The scores obtained from the Bacskó assessment, which are displayed in Table in panel **(B)**. **(C)** Atrioventricular blocks (AVB), in minutes (min); **(D)** isolated ventricular extrasystoles (VE), in units (uni); **(E)** Ventricular tachycardias (VT), in minutes (min). Data are expressed as mean ± SD values. Differences were analyzed using two-way ANOVA followed by Tukey’s *post hoc*. *p* < 0.05 was significant (*p < 0.05 vs. Sham; ^#^ *p* < 0.05 vs. Sham-Hg; ^+^ *p*< 0.05 vs. MI).

Electrocardiogram recordings (Figure 4) showed an increase in atrioventricular blocks in MI groups (MI and MI-Hg) compared to Sham (Figure 4C). Moreover, ventricular extrasystoles (Figure 4D), ventricular tachycardia (Figure 4E), and Basckó coefficient (Figure 4A) revealed that arrhythmias after MI were aggravated by Hg exposure. The severity of arrhythmias, as classified by the Basckó score, is showed in panel B (Figure 4B).

In addition, analysis of ECG tracing revealed an increase in R amplitude in MI groups when compared with the Sham group. Additionally, there was an increase in S and T amplitude, as well as ST height, in both the MI and MI-Hg groups compared to the Sham and Sham-Hg group (Table 2).

**TABLE 2:** Summary of the electrocardiogram data.

	Period	Sham (*n* = 21)	Sham-Hg (*n* = 20)	MI (*n* = 37)	MI-Hg (*n* = 40)
HR (bpm)	Pre-surgery	283 ± 35.41	265 ± 51	266 ± 25	269 ± 20
	Post-surgery	274 ± 43	264 ± 58	236 ± 53*	257 ± 55
AVB (min)	Pre-surgery	0 ± 0	0 ± 0	0 ± 0	0 ± 0
	Post-surgery	0 ± 0	0.81 ± 3.01	4.35 ± 5.82*^#^	3.64 ± 5.53*^#^
VE (uni)	Pre-surgery	0 ± 0	0 ± 0	0 ± 0	0 ± 0
	Post-surgery	4.24 ± 18.97	19.70 ± 48.16	89.13 ± 95.78*^#^	136.59 ± 114.12*^#+^
VT (min)	Pre-surgery	0 ± 0	0 ± 0	0 ± 0	0 ± 0
	Post-surgery	0 ± 0.01	0.36 ± 1.21	1.67 ± 1.88*^#^	3.26 ± 2.95*^#+^
Baczkó (score)	Pre-surgery	0 ± 0	0 ± 0	0 ± 0	0 ± 0
	Post-surgery	0.24 ± 0.89	0.75 ± 1.59	2.97 ± 1.83*^#^	4.00 ± 1.32*^#+^
PRi (ms)	Pre-surgery	0.04 ± 0.01	0.04 ± 0.01	0.04 ± 0.01	0.05 ± 0.01
	Post-surgery	0.04 ± 0.01	0.04 ± 0.01	0.05 ± 0 .01	0.05 ± 0.01
P (ms)	Pre-surgery	0.02 ± 0.01	0.03 ± 0.01	0.03 ± 0.01	0.02 ± 0.01
	Post-surgery	0.03 ± 0.01	0.03 ± 0.01	0.03 ± 0	0.03 ± 0
QRSi (ms)	Pre-surgery	0.02 ± 0.01	0.02 ± 0.01	0.02 ± 0.01	0.02 ± 0.01
	Post-surgery	0.02 ± 0.01	0.02 ± 0.01	0.02 ± 0.01	0.03 ± 0 .01
QTi (ms)	Pre-surgery	0.06 ± 0.02	0.06 ± 0.02	0.05 ± 0.02	0.06 ± 0.02
	Post-surgery	0.06 ± 0.02	0.06 ± 0.03	0.05 ± 0.01	0.05 ± 0.01
JTi (ms)	Pre-surgery	0.02 ± 0.02	0.03 ± 0.01	0.03 ± 0.01	0.03 ± 0.02
	Post-surgery	0.03 ± 0.01	0.03 ± 0.01	0.03 ± 0.01	0.03 ± 0.01
P Amp (mV)	Pre-surgery	0.02 ± 0.01	0.02 ± 0.02	0.02 ± 0.03	0.02 ± 0.03
	Post-surgery	0.02 ± 0.02	0.02 ± 0.02	0.02 ± 0.02	0.02 ± 0.02
Q Amp (mV)	Pre-surgery	0 ± 0.01	0 ± 0.01	0 ± 0.01	0 ± 0.01
	Post-surgery	0.01 ± 0.01	0 ± 0.02	−0.01 ± 0.02	0 ± 0.02
R Amp (mV)	Pre-surgery	0.17 ± 0.01	0.16 ± 0.01	0.18 ± 0.01	0.19 ± 0.01
	Post-surgery	0.15 ± 0.01	0.23 ± 0.02	0.33 ± 0.02*	0.33 ± 0.02*
S Amp (mV)	Pre-surgery	0.03 ± 0.01	−0.04 ± 0.04	−0.02 ± 0.11	−0.02 ± 0.10
	Post-surgery	0 ± 0.01	0 ± 0.09	0.13 ± 0.12*^#^	0.10 ± 0.11*^#^
T Amp (mV)	Pre-surgery	0.10 ± 0.10	0.07 ± 0.13	0.09 ± 0.15	0.18 ± 0.26
	Post-surgery	0.10 ± 0.10	0.15 ± 0.21	0.42 ± 0.25*^#^	0.39 ± 0.23*^#^
ST Height (mV)	Pre-surgery	0.03 ± 0.06	0.01 ± 0.06	0 ± 0.04	0 ± 0.05
	Post-surgery	0.09 ± 0.11	0.15 ± 0.21	0.39 ± 0.20*^#^	0.37 ± 0.20*^#^

HR, heart rate; bpm, beats per minute; AVB, atrioventricular blocks; min, minutes; VE, ventricular extrasystoles; uni, units; VT, ventricular tachycardia; i, interval; mV, millivolt; Amp, amplitude. The results are reported as mean ± SD. Differences were analyzed using two-way ANOVA followed by Tukey’s *post hoc* test. *p* < 0.05 was considered significant: *p < 0.05 vs. Sham, ^#^ *p* < 0.05 vs. Sham-Hg; ^+^ *p* < 0.05 vs. MI.

### 3.4 Ponderal data

After 4 weeks of treatment, when we reached the endpoint of the study, it was evaluated the ponderal parameters. No significant changes were observed in the body weight of the Sham and Sham-Hg groups. However, the MI-Hg group showed a decrease in body weight compared to both the Sham and Sham-Hg groups, although this difference did not reach statistical significance when compared to the MI group.

The MI and MI-Hg groups exhibited a reduced total weight gain compared to the Sham and Sham-Hg groups. The heart weight and lung weight normalized to body weight ratio (HW/BW and LW/BW, respectively) were increased in the MI and MI-Hg groups compared to the Sham and Sham-Hg groups. However, the lung weight to body weight ratio (LW/BW) showed a greater increase in the MI group compared to the MI-Hg group. The LW/BW ratio is indicative of pulmonary congestion. It is likely that the animals that survived in the MI-Hg group experienced a lower degree of pulmonary congestion, which may have aided in their survival despite exposure to mercury at this specific time point (Table 3).

**TABLE 3:** Ponderal data.

	Sham (*n* = 18)	Sham-Hg (*n* = 10)	MI (*n* = 15)	MI-Hg (*n* = 14)
Initial BW (g)	263.9 ± 10.1	266.1 ± 8.0	266.9 ± 8.5	262,0 ± 9.6
BW at the endpoint (g)	361.7 ± 31.2	357.0 ± 40.9^“^	341.1 ± 23.8^“^	332,9 ± 42.5^“^*^#^
Total weight gain (g)	98.3 ± 20.2	99.2 ± 27.5	74.2 ± 28*^#^	77.2 ± 30.9*^#^
HW/BW (mg/g)	2.82 ± 0.19	2.87 ± 0.45	3.83 ± 0.84*^#^	3., 73 ± 0.81*^#^
RV/BW (mg/g)	0.61 ± 0.18	0.65 ± 0.13	0.87 ± 0.22*^#^	0.76 ± 0.24*
LW/BW (mg/g)	5.11 ± 0.84	4.69 ± 0.65	8.97 ± 2.36*^#^	7.50 ± 2.59*^#+^

Initial body weight, final body weight, total gain of weight in grams (g). Heart weight to body weight ratio (HW/BW), right ventricle to body weight ratio (RV/BW), and lung weight to body weight ratio (LW/BW) in mg/g. The results are reported as the mean ± SD. Statistical analyses were evaluated by two-way ANOVA followed by Tukey’s *post hoc*. *p* < 0.05 was significant: “*p* < 0.05 versus initial body weight; *p < 0.05 vs. Sham, ^#^ *p* < 0.05 vs. Sham-Hg; ^+^ *p* < 0.05 vs. MI.

### 3.5 Hemodynamic parameters after 4 weeks of survival

Sham-Hg group showed an increase in both systolic and diastolic blood pressure. MI and MI-Hg groups displayed a decrease in systolic blood pressure, left ventricle systolic pressure, and +dP/dt and −dP/dt values. LVEDP was increased in the MI group compared to MI-Hg, Sham and Sham-Hg groups. The MI-Hg group exhibited LVEDP levels similar to those of the Sham and Sham-Hg groups. On the other hand, the DBP was reduced only in the MI-Hg group as shown in Table 4.

**TABLE 4:** Hemodynamic parameters.

	Sham (*n* = 13)	Sham-Hg (*n* = 13)	MI (*n* = 8)	MI-Hg (*n* = 9)
HR (bpm)	260.89 ± 54.47	246.24 ± 48.30	251.40 ± 17.80	252.98 ± 52.58
SBP (mmHg)	105.08 ± 10.31	114.71 ± 12.84*	95.47 ± 10.14*^#^	90.01 ± 8.89*^#^
DBP (mmHg)	76.13 ± 11.90	89.23 ± 15.11*	78.82 ± 3.82	63.60 ± 12.81*^#+^
LVSP (mmHg)	113.96 ± 18.72	118.3 ± 16.60	98.61 ± 8.31*^#^	91.70 ± 12.12*^#^
LVEDP (mmHg)	7.31 ± 4.13	5.38 ± 4.57	15.95 ± 8.02*^#^	9.56 ± 3.89^+^
+dP/dt (mmHg/s)	5514.56 ± 1881.21	5779.31 ± 2413.98	3623.05 ± 890.60*^#^	2782.07 ± 910.75*^#^
−dP/dt (mmHg/s)	−3500.43 ± 1395.55	−3179.75 ± 1117.58	−2247.60 ± 345.34*^#^	−1832.74 ± 352.62*^#^

SBP, Systolic blood pressure; DBP, diastolic blood pressure; LVSP, left ventricle systolic pressure; LVEDP, left end-diastolic pressure; and positive (+dP/dt) and negative (−dP/dt) rates of pressure development in the left ventricle (LV). The results are reported as the mean ± SD. Differences were analyzed using two-way ANOVA followed by Tukey’s *post hoc* was used. *p* < 0.05 was significant: *p < 0.05 vs. Sham, ^#^ *p* < 0.05 vs. Sham-Hg; ^+^ *p* < 0.05 vs. MI.

### 3.6 Reactive oxygen species

The Oxidative stress represents an imbalance between ROS production and the cellular antioxidant defense system.

To evaluate whether mercury exposure alters the cardiac production of reactive oxygen species (ROS), we measured their levels in the papillary muscles of the same rats that underwent all the analyses in this study.

**FIGURE 5:**
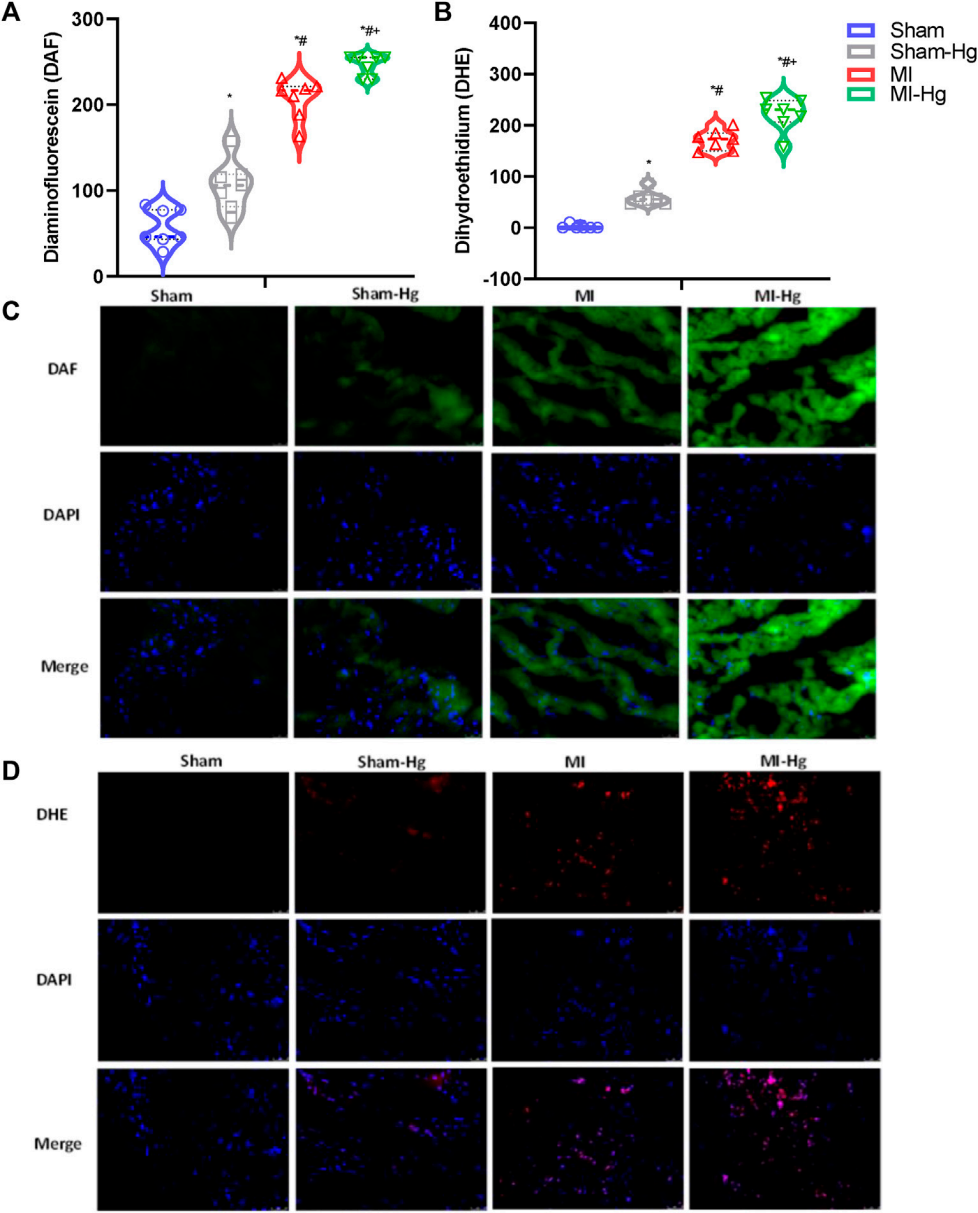
Exposure to Hg resulted in an increase in ROS generation. The MI-Hg group exhibited significantly higher levels of ROS generation. Fluorescence microscopy images of transverse sections collected from the posterior papillary muscle of the LV were obtained after incubation with Diaminofluorescein (DAF) [Panel **(A)**] to detect Nitric Oxide (NO) and Dihydroethidium (DHE) [Panel **(B)**] to detect Anion Superoxide (O^−^_2_). Representative fluorographs analysis of papillary muscles labeled with the NO-sensitive fluorescent dye DAF [Panel **(C)**] and DHE [Panel **(D)**]. Scale bar 100 mm (×40 magnification). Data expressed as mean ± SD. Differences were analyzed using 2-way ANOVA, followed by Tukey’s *post hoc* test. *p* < 0.05 was considered significant. *p < 0.05 vs. Sham; ^#^ *p* < 0.05 vs. Sham-Hg, ^+^ *p* < 0.05 vs. MI.

Our results demonstrated that *in situ* nitric oxide (NO) and superoxide anions levels were increased in the MI and MI-Hg groups compared to the Sham and Sham-Hg groups. As expected, the exposure to mercury resulted in increased ROS levels in the Sham-Hg and MI-Hg groups. Furthermore, as expected, the occurrence of MI also increased NO and superoxide anions levels. When both factors, MI and Hg exposure, were combined, the levels of NO and superoxide anions showed a further increase, as observed in the MI-Hg group (Table 5; Figure 5).

**TABLE 5:** Diaminofluorescein (DAF) to identified levels of nitric oxide (NO) and Dihydroethidium (DHE) to identified levels of superoxide anion (O_2_^−^), expressed in arbitrary units (a.u).

	Sham (*n* = 7)	Sham-Hg (*n* = 7)	MI (*n* = 7)	MI-Hg (*n* = 7)
DAF	57.27 ± 21.29	106.3 ± 29.01*	206.98 ± 23.46*^#^	246.1 ± 12.02*^#+^
DHE	1.79 ± 4.39	58.88 ± 14.71*	170.88 ± 19.21*^#^	220.76 ± 32.13*^#+^

The results are reported as mean ± SD. The number of rats (*n*) included in each group is indicated in parenthesis. For statistical analysis, two-way ANOVA followed by Tukey’s *post hoc* test was used. *p* < 0.05 was considered significant: *p < 0.05 vs. Sham; ^#^ *p* < 0.05 vs. Sham-Hg, ^+^ *p* < 0.05 vs. MI.

## 4 Discussion

### 4.1 Mercury toxicity and myocardial infarction mouse model

Mercury (Hg) exposure remains a major problem to the population, which requires action for proper control (GMA, 2018).

Hg compounds can be categorized into three classes: elemental mercury, inorganic mercury compounds (e.g., HgCl_2_) and organic mercury compounds (e.g., methylmercury). Each mercury class has distinct chemical properties that contribute to different toxicokinetics and toxicodynamics (Atsdr, 2022). Exposures to elemental mercury may affect the nervous system and exposures to inorganic mercury compounds may affect the kidneys. Exposures to methylmercury are associated with adverse effects on brain development. Studies on the toxicity of methylmercury carried out over recent decades have provided a growing body of evidence that chronic, relatively low-level methylmercury exposures can be associated with a range of other adverse health outcomes as well, affecting, for example, the cardiovascular system (Genchi, 2017).

Several studies have linked Hg exposure to an increased risk of atherosclerosis, hypertension, coronary dysfunction and MI (Salonen et al., 1999; Guallar et al., 2002; Genchi, 2017). However, there is no consensus on the dose-response relationship between Hg exposure and cardiotoxicity.

In the present study the MI outcomes were exacerbated by Hg exposure. It was used a concentration below the threshold value of 15 μg/L in the blood recommended by WHO. Our results showed that Hg exposure was associated with a worse prognosis after MI by increasing the incidence of arrhythmias, and elevating levels of ROS, observed in the MI-Hg group.

MI surgically-induced model was performed to induce transmural infarction between 40% and 60% of the left ventricular surface without damaging the interventricular septum. This experimental model is the most widely used to study the remodeling process of ventricles after MI injury, which leads to congestive heart failure syndrome. The manifestations observed in rats post-MI, such as myocardial necrosis, effectively reproduce the findings in humans with cardiac decompensation supporting the study of the pathogenesis, pathophysiology, and treatment of congestive heart failure (Tucci, 2011). In our experimental design other associated diseases were excluded, making it easier to determine the combined effect of mercury toxicity and MI. Therefore, experimental model of MI allows us to make significantly more controlled observations and overcome the limitations associated with comorbidity that are often seen in clinical studies. Additionally, our study showed similar scar sizes between groups (MI and MI-Hg) which allows for more homogeneous analysis of the data. Furthermore, Hg exposure was controlled by administering the first dose of 4.34 μg/kg and daily doses of 0.07 μg/kg to reach a plasma concentration of about 29 nM (7.97 ± 0.59 ng/mL) at the end of the study which closely corresponds the levels observed in humans exposed to mercury. This dose was calculated using the volume of distribution multiplied by the plasmatic concentration. Considering that Hg is distributed equally throughout the body, its distribution was considered equal to the total water content in the body (80%). It has been clearly demonstrated that Hg can produce cardiotoxicity.

### 4.2 Mercury effects after myocardial infarction

Our study demonstrated that 7 days after a MI, the group exposed to Hg had a higher mortality rate compared to the group receiving saline treatment. 31.82% of the rats in the MI-Hg group died, with 22.74% dying within 24 h, 4.54% within 48 h, and another 4.54% within 72 h after the MI procedure. In contrast, 21.43% of the rats in the MI group died within 24 h of surgery, but this percentage did not change during the first 72 h. The sham group, whether exposed or not to Hg, survived until the end of the study period. The higher mortality in MI-Hg group suggests that Hg toxicity may have a worse negative impact on the outcome of a MI. We correlated arrhythmic events with mortality and found a strong correlation in all arrhythmia analyses (*r* > 0.7; Figure 3B).

A meta-analysis study conducted among the Inuit population in Canada showed that exposure to methylmercury might impact heart rate variability, which could be the result of a decrease in parasympathetic activity (Valera et al., 2013). The methylmercury could influence the heart rate via the parasympathetic activity of the autonomic nervous system slowing the heart rate. On the other hand, the methylmercury exposure has increased noradrenaline output responsible for the acceleration of the heart rate (Valera et al., 2013). Indeed, heart rate variability is a strong predictor of mortality after MI and it is associated with an increased mortality in patients post MI due to occurrence of arrhythmic events (Reed et al., 2005; Pizzo et al., 2022).

Although we did not assess heart rate variability, we found that arrhythmic events in the MI-Hg group were more strongly linked to mortality. While arrhythmias are expected after an MI, those that occurred after exposure to even low concentrations of Hg following the MI were more pronounced. Clearly, even low concentrations of Hg worsen the hemodynamic repercussions after MI.

### 4.3 Blood pressure changes after mercury exposition followed by myocardial infarction

Literature reports on the association between Hg exposure and increased systolic and diastolic blood pressure levels are controversial (Hu et al., 2018). Our findings demonstrated an increase in both systolic and diastolic in the Sham-Hg group, while these parameters were decreased in the MI-Hg group. However, the MI-Hg group represents the animals that survived. It has been shown that chronic exposure to Hg increases blood pressure and is associated with increased activity of the renin-angiotensin system (Peçanha et al., 2010; Vassallo et al., 2019). It is also known that exposure to low concentrations of Hg can lead to vascular injury, as it increases peripheral vascular resistance, which in turn is associated with a higher risk of mortality (Lemos et al., 2012). We suggest that the increased mortality observed within 72 h after MI in the MI-Hg group could be attributed to the increased sympathetic activity leading to elevated blood pressure in those rats. Conversely, the surviving rats presented a decrease in systolic and diastolic blood pressure. Therefore, we suggest that the surviving animals in the MI-Hg group, who were exposed to Hg and acute MI for 7 days, had less hemodynamic repercussions. Furthermore, we observed cardiac hypertrophy and pulmonary congestion, which may be attributed to an increase in myocardial afterload. These factors could potentially be influenced by elevated peripheral vascular resistance (Fardin et al., 2019; Vassallo et al., 2020), despite the absence of clear evidence of increased blood pressure in all groups following 30 days of mercury exposure. Additionally, chronic exposure to Hg can induce increased blood pressure through phenylephrine, which is caused by a decrease in NO and an increase in ROS (Fardin et al., 2019; Vassallo et al., 2020; Ronchetti et al., 2022).

### 4.4 Association between arrhythmias and ROS

Previous studies showed that exposure to low concentrations of Hg leads to vascular injury following the increase in peripheral vascular resistance that is associated with a higher risk of mortality (Lemos et al., 2012; Fardin et al., 2019; Simões et al., 2020; Ronchetti et al., 2022). Moreover, the increase of mortality in animals exposed to Hg may be associated with Hg binding to thiol groups of cardiac proteins, inducing maladaptive changes in channels that influence the action potentials and cardiac rhythm (Oliveira et al., 2019; Vassallo et al., 2019).

In this present study, we observed that the combination of ischemic events such as MI and exposure to even low doses of mercury (Hg) is associated with an increased risk of mortality. Indeed, this high mortality was correlated with major arrhythmic events in the group MI-Hg. The relationship between ROS and MI is also well-known and studied for a long time (Moris et al., 2017). In 1998, Chen et al., 1998, demonstrated that the overexpression of the antioxidant enzyme SOD was able to significantly decrease the infarct area of mice.

The increase in the arrhythmias observed in the electrocardiogram of MI-Hg group could be explained by both Hg and infarction, even separately, stimulating inflammation and oxidation (Hori and Nishida, 2008; Tinkov et al., 2015; Faria et al., 2018). Whereas the increase in ROS production causes peroxidation of cell membrane lipids and stimulates the opening of ion channels that induce the release of Ca^2+^ by the sarcoplasmic reticulum, hampering its reuptake. At the same time, reactive species influence the production of inflammatory cytokines that interfere with the intracellular Ca^2+^ balance. Ionic imbalances in myocytes are related to cardiac electrical changes, alterations in contractility and, consequently, to arrhythmias. (Mukherjee et al., 2002; Hori and Nishida, 2008).

To assess the presence of reactive oxygen species, we harvested papillary muscles from rats from all groups 1 week after ECG recordings. We found higher levels of nitric oxide and superoxide anions after MI, as expected. However, when comparing the animals from MI and MI-Hg groups, it was observed that exposure to the metal caused a notable increase of the production of the reactive oxygen species. Previous studies by our group also observed an increase in ROS in aortas and mesenteric arteries of rats chronically exposed to low doses of mercury (Wiggers et al., 2008) associated with altered COX-2-expression leading increases the expression of the enzyme NADPH oxidase, which stimulates the formation ROS (Rizzetti et al., 2017). In addition, mercury binds proteins and induces auto-oxidation of the inner mitochondrial membrane, increasing the production of ROS. The metal also promotes the activation of xanthine oxidase, which is also related to the production of ROS (Houston, 2007; Tinkov et al., 2015; Branco et al., 2017; Genchi, 2017).

Furthermore, ROS stimulates inflammatory cell activation and endothelial dysfunction. Several studies showed that Hg both stimulates the production of ROS and promote the reduction of antioxidant proteins in various organs and systems of animals and humans, promoting a redox imbalance and stimulating cell death (Miller and Woods, 1993; Huang et al., 1996; Mahboob et al., 2001; Reus et al., 2003; Kim and Sharma, 2004; Chen et al., 2005; Houston et al., 2007; Pollack et al., 2012; Santana, et al., 2018; Oliveira et al., 2019).

## Conclusion

Our results suggest that exposure to Hg aggravates the effects of MI, worsening the harmful cardiac events triggered by injury, and this has been associated with major arrhythmias occurrence. We reported increased mortality in rats pre-exposed to Hg and submitted to MI. These findings demonstrate that the Hg toxicity cause worsened outcome in MI. Also, as expected, there was an increase in ROS production due to Hg toxicity. Moreover, exposure to Hg in MI resulted in ROS production further elevated. We can conclude that while rats survived after MI, the toxicity of Hg exacerbated the metabolic conditions due to oxidative stress, which in turn could trigger an inflammatory cascade in cardiovascular disease.

Furthermore, our findings in this study suggest that rats in the MI-Hg group, who survived the MI surgery, did not exhibit elevated blood pressure and may have maintained favorable hemodynamic conditions. This could have played a crucial role in their survival following the MI event even Hg exposure.

The present findings have significant implications for public health. As demonstrate the Hg exposure has a detrimental consequence, especially associated with MI. It is crucial to implement stricter measures to combat Hg contamination in the environment and emphasize that Hg contamination is a substantial public health concern, particularly for individuals vulnerable to cardiovascular diseases.

## Future directions

Hg induces ventricular electrical remodeling that increases the likelihood of cardiac arrhythmias, similar to the effects seen in MI. Both Hg exposure and MI have the potential to promote Ca^2+^ overload through exacerbated β-adrenergic stimulation, which may contribute to the arrhythmogenesis process, as observed in this study.

We propose to conduct a thorough investigation into channels and calcium handling to elucidate the underlying mechanisms by which they are affected when Hg exposure and myocardial infarction co-occur, potentially contributing to increased mortality rates.

### Study limitations

This paper revealed the association between Hg exposure and an increased occurrence of arrhythmogenic events in myocardial infarction, which is further correlated with higher mortality rates. Further studies are needed to better understand of the mechanisms through which Hg may contribute to the potential arrhythmogenic effects immediately following MI.

## Data Availability

The raw data supporting the conclusion of this article will be made available by the authors, without undue reservation.
